# Neutrophils Are Not Less Sensitive Than Other Blood Leukocytes to the Genomic Effects of Glucocorticoids

**DOI:** 10.1371/journal.pone.0044606

**Published:** 2012-09-12

**Authors:** Gaelle Hirsch, Anouk Lavoie-Lamoureux, Guy Beauchamp, Jean-Pierre Lavoie

**Affiliations:** Department of Clinical Sciences, Faculty of Veterinary Medicine, Université de Montréal, Saint-Hyacinthe, Quebec, Canada; University of Tübingen, Germany

## Abstract

**Background:**

Neutrophils are generally considered less responsive to glucocorticoids compared to other inflammatory cells. The reported increase in human neutrophil survival mediated by these drugs partly supports this assertion. However, it was recently shown that dexamethasone exerts potent anti-inflammatory effects in equine peripheral blood neutrophils. Few comparative studies of glucocorticoid effects in neutrophils and other leukocytes have been reported and a relative insensitivity of neutrophils to these drugs could not be ruled out.

**Objective:**

We assessed glucocorticoid-responsiveness in equine and human peripheral blood neutrophils and neutrophil-depleted leukocytes.

**Methods:**

Blood neutrophils and neutrophil-depleted leukocytes were isolated from 6 healthy horses and 4 human healthy subjects. Cells were incubated for 5 h with or without LPS (100 ng/mL) alone or combined with hydrocortisone, prednisolone or dexamethasone (10^−8^ M and 10^−6^ M). IL-1β, TNF-α, IL-8, glutamine synthetase and GR-α mRNA expression was quantified by qPCR. Equine neutrophils were also incubated for 20 h with or without the three glucocorticoids and cell survival was assessed by flow cytometry and light microscopy on cytospin preparations.

**Results:**

We found that glucocorticoids down-regulated LPS-induced pro-inflammatory mRNA expression in both cell populations and species. These drugs also significantly increased glutamine synthetase gene expression in both equine cell populations. The magnitude of glucocorticoid response between cell populations was generally similar in both species. We also showed that dexamethasone had a comparable inhibitory effect on pro-inflammatory gene expression in both human and equine neutrophils. As reported in other species, glucocorticoids significantly increase the survival in equine neutrophils.

**Conclusions:**

Glucocorticoids exert genomic effects of similar magnitude on neutrophils and on other blood leukocytes. We speculate that the poor response to glucocorticoids observed in some chronic neutrophilic diseases such as severe asthma or COPD is not explained by a relative lack of inhibition of these drugs on pro-inflammatory cytokines expression in neutrophils.

## Introduction

Neutrophils play a central role in innate immunity, acting as the first line of host defense against invading organisms. They are the predominant cell type involved in the cellular phase of acute inflammation. Their role in the inflammatory process was once thought to be restricted to phagocytosis and the release of cytotoxic agents, such as superoxide and other reactive oxygen species [1]. It is now known that these cells have also the capacity to synthesize a large number of pro-inflammatory cytokines, chemokines and growth factors, in response to a variety of stimuli [1]. Neutrophil activation leads to the development and maintenance of inflammatory response, but also to the resolution of inflammation by neutralization of the offending insult. These cells are removed from tissues by programmed cell death and phagocytosis, avoiding the release of harmful substances and promoting a return to homeostasis. Thus, a prolonged activation of neutrophils or delayed apoptosis contributes to maintaining chronic inflammation and leads, in turn, to damage to surrounding tissues [2].

Glucocorticoids (GCs) are potent anti-inflammatory drugs used for the treatment of chronic inflammatory and immune conditions. Neutrophils are, however, generally considered less responsive to GCs compared to other inflammatory cells [3], [4]. This is due to the lack of inhibitory effects of these drugs on neutrophils’ degranulation, chemotaxis or release of arachidonic acid metabolites [5]. Furthermore, GCs increase human neutrophil survival [6], [7]. Chronic conditions associated with neutrophilic inflammation, such as severe asthma and chronic obstructive pulmonary disease (COPD), also tend to be clinically resistant to corticotherapy [8], [9], [10], [11], [12], [13]. Conversely, these drugs have also been shown to down-regulate the production of several cytokines and adhesion molecules in rat [14], human [15], [16], [17], bovine [18], [19] and equine [20] neutrophils. These studies question the proposed inherent corticoresistance of neutrophils. Comparative potencies of GCs on neutrophils and other leukocytes have not been thoroughly studied and would be an alternative way to measure sensitivity of neutrophils to these drugs.

Based on these findings, we compared the effects of three different glucocorticoids through gene expression and cell survival assays in equine peripheral blood neutrophils and neutrophil-depleted leukocytes. To determine if the response is species specific, we also studied human leukocytes. We hypothesized that GCs exert effects of similar magnitude on neutrophils and neutrophil-depleted leukocytes in both species.

## Materials and Methods

### Ethics Statement

Six healthy adult mixed-breed mares (weighing 450–500 kg; mean ± SD, 11±2.7 years of age) and 4 healthy non-smoker adult female volunteers (mean ± SD, 28±1.6 years of age) were studied. Mares were part of the Faculty of Veterinary Medicine of the Université de Montréal research herd. All animal experimental procedures were performed in accordance with the guidelines of the Canadian Council on Animal Care and were approved by the Animal Care Committee of the Faculty of Veterinary Medicine of the Université de Montréal (10-Rech-1514). This study was also approved by the Ethic Research Committee of the Faculty of Medicine of the Université de Montréal (Protocol 11-029-CERFM-D) and an informed, written consent was obtained from the healthy human volunteers.

### Neutrophil and Neutrophil-depleted Leukocyte Isolation

Equine and human blood was drawn into heparinized sterile tubes (Groupe Tyco Medical, Pointe-Claire, QC, CA).

#### Equine neutrophil isolation

Neutrophils were isolated from the blood using density gradient and immunomagnetic selection to achieve high cell purity. Briefly, the polymorphonuclear-rich cell layer was harvested following centrifugation of whole blood on a density gradient solution composed of sodium metrizoate and Dextran 500 (Lympholyte®-poly Cell Separation Media, Cedarlane Laboratories, Burlington, ON, CA) according to the manufacturer’s instructions. The remaining red blood cells (RBCs) were lysed by hypotonic treatment. Cells were then resuspended in degassed PBS-buffer (w/o Ca^2+^/Mg^2+^, pH 7.2; 0.5% BSA, Invitrogen, Carlsbad, CA, USA; 2 mM EDTA, Sigma-Aldrich, St. Louis, MO, USA).

Positive immunomagnetic selection (MACS® Cell Separation, Miltenyi Biotec Bergisch Gladbach, GER) was then performed as described previously with minor modifications [21]. Briefly, the polymorphonuclear-rich cell suspension obtained as described above was incubated with a primary monoclonal antibody directed against equine neutrophils (Anti-Thy1, VMRD, Pullman, WA, USA) and a secondary rat anti-mouse IgM antibody conjugated to paramagnetic microbeads (MACS® Cell Separation). The cells were then loaded on a ferromagnetic LS separation column (MACS® Cell Separation) and neutrophils eluted in the positive cell fraction were pelleted and resuspended in degassed-PBS buffer.

#### Equine neutrophil-depleted leukocyte isolation

Neutrophil-depleted leukocyte isolation was performed using negative immunomagnetic selection (MACS® Cell Separation) as described earlier [22]. Briefly, neutrophils were isolated using positive immunomagnetic selection (MACS® Cell Separation) as described above, neutrophil-depleted leukocytes eluted in the negative cell fraction were pelleted and the RBCs were removed with isotonic NH_4_Cl solution (155 mM NH_4_Cl, 10 mM KHC0_3_, 0.1 mM EDTA, pH 7.4, all products from Sigma-Aldrich, St. Louis, MO, USA). Cells were washed twice in degassed-PBS buffer using low speed centrifugation (1000 rpm, 10 min, GS-6R Centrifuge, Beckman, Brea, CA, USA) to remove platelets.

Cytospin slides were prepared (Shandon Cytospin 2, Thermo Scientific now part of Thermo Fisher Scientific, Pittsburgh, PA, USA) and stained with Protocol Hema 3 (Fisher Scientific, now part of Thermo Fisher Scientific, Pittsburgh, PA, USA) for differential counting of ≥400 cells to assess purity. Viability was determined by ADAM automatic Cell Counter (Montreal-Biotech Inc., Montréal, QC, CA). The purity and viability of equine neutrophils were ≥99.5% and ≥95.0% respectively. The purity and viability of neutrophil-depleted leukocytes were ≥91.5% and ≥96.5% respectively.

#### Human neutrophil and neutrophil-depleted leukocyte isolation

Human granulocytes were isolated from whole blood using dextran sedimentation and Ficoll-Paque™ gradient (Ficoll-Paque™ Premium 1.084, GE Healthcare Biosciences, Uppsala, SE) as described previously [23] with minor modifications. Neutrophil-depleted leukocyte layer was harvested following whole blood centrifugation on Ficoll-Paque™ gradient, resuspended in degassed-PBS buffer and kept on ice for 1 h until further use. Granulocyte pellet was resuspended in degassed-PBS buffer after RBCs were removed with isotonic NH_4_Cl solution.

Neutrophil positive immunomagnetic selection by MACS® was then performed according to the manufacturer’s instructions. Briefly, neutrophils were obtained from the granulocyte suspension by incubation with a monoclonal mouse anti-human CD16 antibody conjugated to paramagnetic microbeads (MACS® Cell Separation) before being loaded on a ferromagnetic LS separation column (MACS® Cell Separation). Neutrophils eluted in the positive cell fraction were pelleted and resuspended in degassed-PBS buffer. The purity and viability of human neutrophils were ≥99.7% and ≥91.5% respectively. The purity and viability of human mononuclear cells were ≥93.5% and ≥97.5% respectively.

### Cell Culture

After isolation, cells were washed twice and resuspended in complete culture medium at 5×10^6^ cells/mL for neutrophils and between 2 to 5×10^6^ cells/mL for neutrophil-depleted leukocytes. Components of medium were RPMI 1640 supplemented with 10% heat inactivated low-endotoxin FBS, 2 mM _L_-glutamine, 100 U/mL penicillin and 100 µg/mL streptomycin (all products from GIBCO®, Invitrogen, Carlsbad, CA, USA). Cells were cultured in 6- or 12-well plates (non-treated plastic, UltiDent Scientific, St. Laurent, QC, CA) in the presence or absence of lipopolysaccharide (LPS) from Escherichia coli 0∶111B4 (100 ng/mL in Dulbecco’s PBS) alone or combined with hydrocortisone, prednisolone or dexamethasone (10^−8^ M and 10^−6^ M in ethanol) (all products from Sigma-Aldrich, St. Louis, MO, USA). Cells were incubated at 37°C in a 5% CO_2_ atmosphere for 5 h. Cell viability was assessed before homogenization in TRIzol® reagent (Invitrogen, Carlsbad, CA, USA).

### RNA Extraction and Reverse Transcription

Total cellular RNA extraction was performed according to the manufacturer’s instructions using a three-step nucleic acid precipitation with 0.2 volume of chloroform, 1 volume of isopropanol and 75% ethanol (TRIzol® reagent; Invitrogen, Carlsbad, CA, USA). RNA pellets were air-dried, and total RNA concentration and purity were evaluated by spectrophotometry (NanoDrop 2000, Thermo Scientific®, Thermo Fisher Scientific, Waltham, MA, USA). Five hundred nanograms of total RNA were retro-transcribed into cDNA as described elsewhere [24].

### Quantitative PCR

Quantitative PCR (qPCR) reactions were performed with the Rotor-Gene RG-3000 (Corbett Research, Sydney, AS) as previously described with minor modifications [24]. One microliter of cDNA template was added to the QuantiTect® SYBR® Green PCR Master Mix (Qiagen, Toronto, ON, CA) in a 20-µL final PCR volume containing 0.5 µM each sense and antisense primers and MgCl_2._ Equine and human specific primers (Table S1) were designed to span exon-intron boundaries to prevent amplification of genomic DNA. qPCR conditions were optimized for all primer sets. Amplification conditions included a denaturation step of 10 min at 95°C followed by a maximum of 40 cycles of denaturation, annealing and elongation steps. Each reaction was run in duplicate with an appropriate negative control. All concentrations of target gene cDNA were calculated relatively to their respective standard curves. Absolute values were corrected using glyceraldehyde 3-phosphate dehydrogenase (GAPDH) as a reference gene. Gene expression was reported as the relative variation (fold increase) to unstimulated cell mRNA levels (arbitrary value of 1).

### Flow Cytometry

#### Equine neutrophil isolation and culture

Equine neutrophils were isolated from whole blood using a density gradient centrifugation technique (Lympholyte®-poly Cell Separation Media) according to the manufacturer’s instructions. The remaining RBCs were lysed by short treatment of the pellet fraction with isotonic NH_4_Cl solution. As a subsequent neutrophil positive immunomagnetic selection by MACS affected viability, this step was not performed. Neutrophils purity and viability were ≥93.5% and ≥98.5% respectively. After isolation, equine neutrophils were washed twice and resuspended at 1×10^6^ cells/mL in complete culture medium. Cells were then incubated at 37°C in a 5% CO_2_ atmosphere for 20 h in the presence of hydrocortisone, prednisolone or dexamethasone (10^−8^ M and 10^−6^ M) or their vehicle (ethanol) as a negative control.

#### Quantification of neutrophil survival

After 20 h of culture, neutrophil count was determined by ADAM automatic Cell Counter (Montreal-Biotech Inc., Montréal, QC, CA). As cell loss appeared more marked in unstimulated cells compared to GC-treated neutrophils, this factor was taken into account when calculating the percentages of viable and apoptotic neutrophils. Neutrophil survival was assessed by flow cytometry. Cells were washed twice with cold Dulbecco’s PBS (Invitrogen, Carlsbad, CA, USA) and resuspended in binding buffer?at a concentration of 10^7^ cells/mL. One hundred microliters of the cell suspension was incubated with 5 µL of APC Annexin V, an apoptosis cell marker and 5 µL of 7-Aminoactinomycin D (7-AAD), a necrosis cell marker (BD Pharmingen™, San Diego, CA, USA) for 15 min at room temperature in the dark. Finally, 400 µL of binding buffer was added to each tube. Cells were placed on ice and analyzed within 1 h by flow cytometry. Data were collected from 10,000 events gated on granulocytes and analyzed using CellQuest Pro software on a FACScalibur instrument (BD Biosciences, Mississauga, ON, CA). Unstained cells as well as single marker-stained cells were used to set photomultipliers voltage and compensation parameters for fluorescence detection in FL-3 and FL-4 channels. APC Annexin V-negative and 7-AAD-negative cells were considered viable.

Neutrophil survival was also determined using light microscopy analysis. Briefly, after 20 h of culture, neutrophils were sedimented by cytocentrifugation on a glass microscope slide and stained with Protocol Hema 3 method. One blinded investigator assessed the percentage of viable and apoptotic neutrophils on cytospin preparations by analysis of 500 cells per slide. Identification of nuclear changes such as condensation of chromatin, simplification of nuclear structure and cytoplasmic vacuolation were considered characteristic of apoptosis and allowed distinction between viable and apoptotic cells [25].

### Statistical Analysis

Data obtained from qPCR and flow cytometry survival experiments were analyzed using repeated-measures linear models. Treatment served as a within-subject factor for each equine cell population, which was analyzed separately. A priori contrasts were done between pairs of treatment means adjusting comparison-wise alpha levels using the sequential Bonferroni correction procedure to ensure that the family-wise error rate remained at the nominal level of 0.05. Gene expression between unstimulated cells and LPS-stimulated cells in equine and human cell populations was compared using a paired t-test. A paired t-test was also used to compare gene expression between treatments within neutrophils and neutrophil-depleted leukocytes in both species and between both doses of each GC within equine neutrophils. Dexamethasone percentage of inhibition of mRNA expression was compared between equine and human neutrophils using a two-sample t-test for unequal variances. Unilateral tests were used when testing predictions. Finally, neutrophil survival assessed by flow cytometry and light microscopy was compared using a mixed linear model with individual as a random factor. As variation between horses (1.8%) proved negligible, the random effect was ignored and a simple linear regression was performed.

## Results

### Glucocorticoids Exert Dose-dependent Effects on Gene Expression in Equine Neutrophils

**Figure 1:**
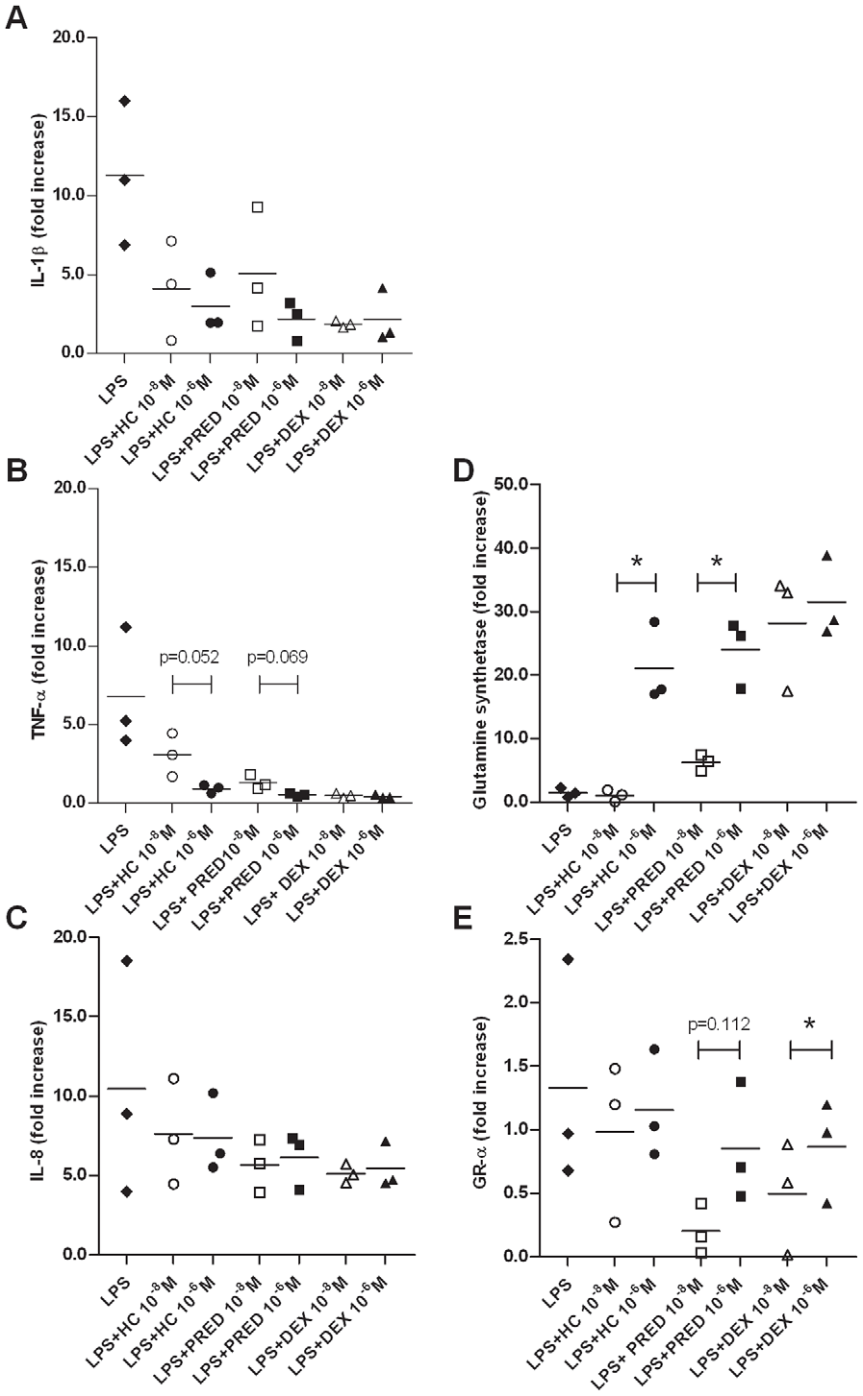
Concentration-dependent effects of glucocorticoids on gene expression in equine peripheral blood neutrophils. Neutrophils were isolated from the blood of 3 healthy horses and were incubated for 5 h with or without lipopolysaccharide (100 ng/mL) combined with hydrocortisone (HC), prednisolone (PRED), dexamethasone (DEX) at 10^−8^ M and 10^−6^ M. Following culture, mRNA expression of pro-inflammatory cytokines (IL-1β, TNF-α and IL-8; A, B and C), glutamine synthetase (D) and GR-α (E) was quantified by qPCR. Absolute values were corrected using GAPDH as a reference gene. Gene expression was reported as the relative variation (fold increase) to unstimulated cell mRNA levels (arbitrary value of 1). Bars represent means. *Significant difference between treatments (p≤0.020).

To assess whether GCs exert a dose-dependent effect on mRNA expression, equine neutrophils were incubated in the presence of LPS alone or combined with hydrocortisone, prednisolone or dexamethasone at 10^−8^ M and 10^−6^ M (n = 3). The mRNA expression of genes that are either repressed (interleukin-1beta, IL-1β; tumor necrosis factor alpha, TNF-α; interleukin-8, IL-8; and glucocorticoid receptor-alpha, GR-α) or induced (glutamine synthetase) by GCs was evaluated using qPCR (Figure 1).

Although there was a trend for a greater inhibitory effect of prednisolone and hydrocortisone on TNF-α mRNA expression at 10^−6^ M compared to 10^−8^ M, this difference was not significant (p = 0.069 and p = 0.052 respectively; Figure 1.B). All three GCs exerted a similar inhibitory effect at both concentrations on IL-1β and IL-8 mRNA expression (Figure 1.A and 1.C). A significant dose- effect on glutamine synthetase mRNA expression was demonstrated for hydrocortisone (p = 0.020) and prednisolone (p = 0.009; Figure 1.D). Unexpectedly, dexamethasone down-regulated GR-α mRNA expression in equine neutrophils, at 10^−8^ M only (p = 0.003). There was also a trend for a greater inhibitory effect of prednisolone on GR-α mRNA expression at the lowest dose compared to 10^−6^ M (p = 0.112; Figure 1.E).

### Glucocorticoids Exert Effects on Gene Expression in Neutrophils and Neutrophil-depleted Leukocytes in Both Species

**Figure 2:**
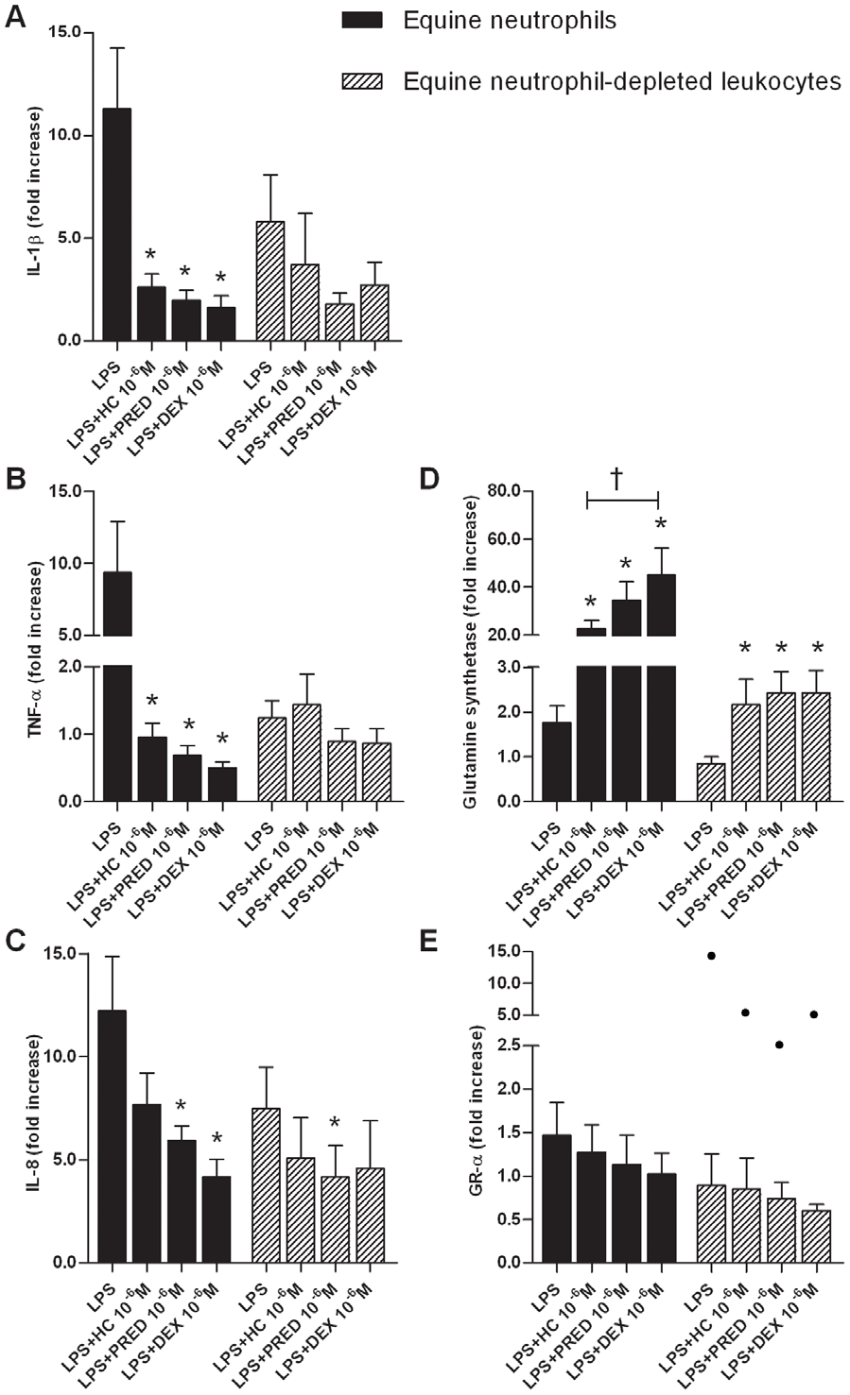
Glucocorticoid effects on gene expression in equine peripheral blood neutrophils and neutrophil-depleted leukocytes. Neutrophils and neutrophil-depleted leukocytes were isolated from the blood of 6 healthy horses and were incubated for 5 h with or without lipopolysaccharide (LPS; 100 ng/mL) alone or combined with hydrocortisone (HC), prednisolone (PRED), dexamethasone (DEX) at 10^−6^ M. Following culture, mRNA expression of pro-inflammatory cytokines (IL-1β, TNF-α and IL-8; A, B and C), glutamine synthetase (D) and GR-α (E) was quantified by qPCR. Absolute values were corrected using GAPDH as a reference gene. Gene expression was reported as the relative variation (fold increase) to unstimulated cell mRNA levels (arbitrary value of 1). Bars represent means + SEM. *Significant effect of treatment over LPS-stimulated cells (p≤0.014). †Significant difference between treatments (p = 0.009).

We next compared the inhibitory effects of GCs on LPS-induced mRNA expression of pro-inflammatory cytokines in equine neutrophils and neutrophil-depleted leukocytes using 10^−6^ M dose (Figure 2.A, 2.B and 2.C). We observed a significant LPS-induced increase in IL-1β (10-fold increase, p≤0.045) and IL-8 (5-fold increase, p≤0.012) mRNA expression in both cell populations. LPS significantly stimulated TNF-α mRNA expression in equine neutrophils only (10-fold increase, p = 0.033). All three GCs down-regulated the LPS-induced IL-1β (p≤0.001), TNF-α (p≤0.004) and IL-8 (p≤0.010) mRNA expression in equine neutrophils except for hydrocortisone, for which the effect on IL-8 expression did not reach significance after Bonferroni correction (p = 0.050). As for neutrophil-depleted leukocytes, prednisolone significantly down-regulated the LPS-induced IL-8 mRNA expression (p = 0.006). Hydrocortisone and dexamethasone also inhibited IL-8 mRNA expression, but to a lesser extent (p = 0.036 and p = 0.014 respectively, not statistically significant after Bonferroni correction).

We also assessed the transactivating effect of GCs on glutamine synthetase mRNA expression in equine neutrophils and neutrophil-depleted leukocytes (Figure 2.D). All three GCs significantly induced glutamine synthetase mRNA expression within both cell populations (p≤0.014). Hydrocortisone had a weaker effect on glutamine synthetase mRNA expression compared to dexamethasone in equine neutrophils (p = 0.009).

Finally, the effect of GCs on their cytosolic receptor mRNA expression was studied. The reduced GR-α mRNA expression caused by dexamethasone in neutrophils was not significant (p = 0.012, not statistically significant after Bonferroni correction; Figure 2.E). LPS had no significant effect on glutamine synthetase and GR-α mRNA expression.

**Figure 3:**
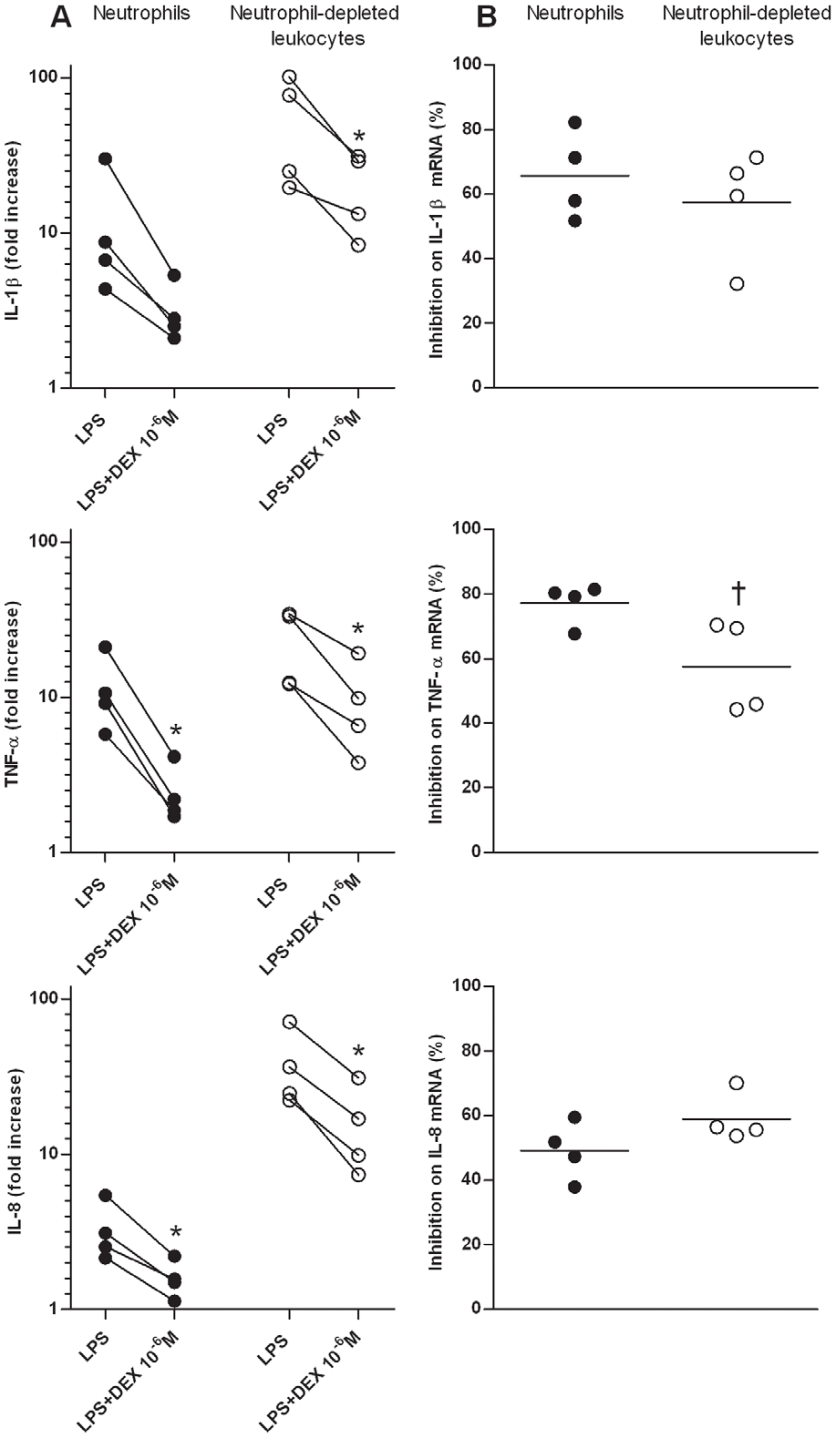
Glucocorticoid effects on pro-inflammatory cytokines gene expression in human peripheral blood neutrophils and neutrophil-depleted leukocytes. Neutrophils and neutrophil depleted leukocytes were isolated from the blood of 4 healthy human volunteers and were incubated for 5 h with or without lipopolysaccharide (LPS; 100 ng/mL) alone or combined with dexamethasone (DEX) at 10^−6^ M. Following culture, mRNA expression of proinflammatory cytokines (IL-1β, TNF-α and IL-8) was quantified by qPCR. Absolute values were corrected using GAPDH as a reference gene. A. Gene expression was reported as the relative variation (fold increase) to unstimulated cell mRNA levels (arbitrary value of 1). Bars represent means. *Significant effect of treatment over LPS-stimulated cells (p≤0.050). B. Dexamethasone percentage of inhibition on LPS-induced mRNA expression was compared between cell populations. A 100% inhibition means that mRNA expression returned to unstimulated cell basal mRNA level. Bars represent means. †Significant effect of treatment between cell populations (p = 0.048).

In order to evaluate a species-specific effect of GCs, human neutrophil and neutrophil-depleted leukocyte response to dexamethasone (10^−6^ M, n = 4) was similarly studied (Figure 3.A). LPS increased IL-8 (p≤0.026) and TNF-α mRNA expression (p≤0.024) in both cell populations, while IL-1β mRNA expression was significantly increased in human neutrophil-depleted leukocytes only (p = 0.036). Dexamethasone down-regulated IL-8 (p≤0.025) and TNF-α (p≤0.022) mRNA expression within each cell population. However, despite a reduced IL-1β mRNA expression caused by dexamethasone within neutrophils in all human volunteers, this effect was not statistically significant (p = 0.086).

### These Effects were Shown to be Similar between Cell Populations and between Species

**Figure 4:**
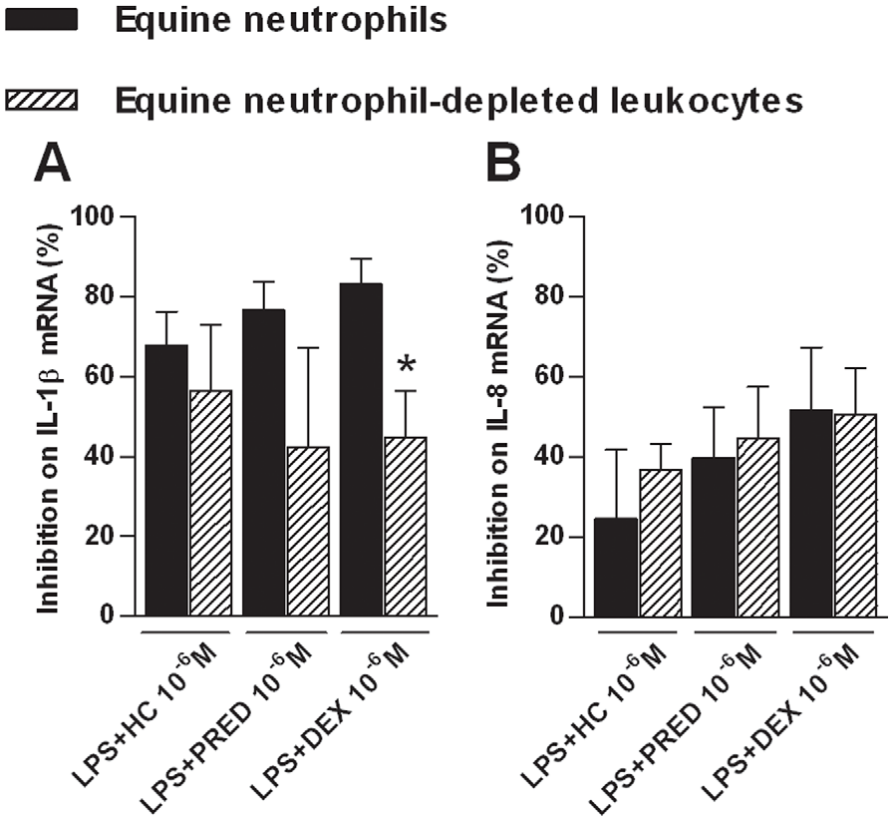
Comparison of glucocorticoid inhibitory effects between equine cell populations. Neutrophils and neutrophil depleted leukocytes were isolated from the blood of 6 healthy horses and were incubated for 5 h with or without lipopolysaccharide (LPS; 100 ng/mL) alone or combined with hydrocortisone (HC), prednisolone (PRED), dexamethasone (DEX) at 10^−6^ M. Following culture, mRNA expression of pro-inflammatory cytokines (IL-1β and IL-8; A and B) was quantified by qPCR. Absolute values were corrected using GAPDH as a reference gene. GC percentage of inhibition on LPS-induced mRNA expression was compared between cell populations. A 100% inhibition means that mRNA expression returned to unstimulated cell basal mRNA level. Bars represent means + SEM. *Significant effect of treatment between cell populations (p = 0.046).

The inhibitory and transactivating effects of GCs on gene expression in neutrophils and neutrophil-depleted leukocytes of equine and human subjects were compared. IL-1β (p = 0.046; Figure 4A) and TNF-α (p = 0.048; Figure 3.B) mRNA expression in equine and human neutrophils respectively, was more strongly inhibited by dexamethasone than in other blood leukocytes. Moreover, the three drugs had also a greater transactivating effect on glutamine synthetase mRNA expression in equine neutrophils compared to neutrophil-depleted leukocytes (from a 20- to 50-fold increase in neutrophils compared to a 2-fold increase in other leukocytes; p≤0.007; Figure 2.D). Equine TNF-α and GR-α mRNA expression was not studied as there was neither effect of LPS nor effect of GCs on both cell populations. Except for genes mentioned above, GCs exerted effects of similar magnitude in both cell populations for both species.

**Figure 5:**
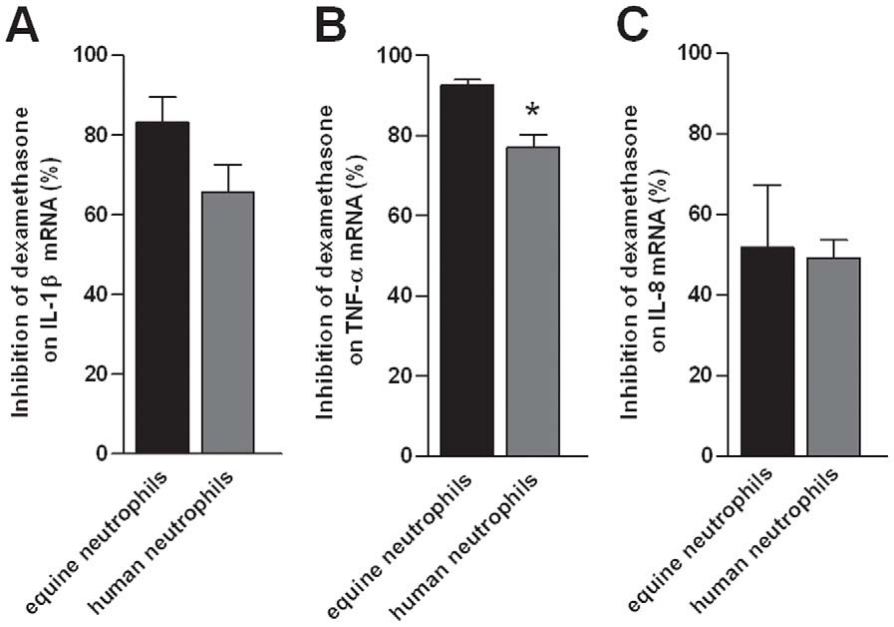
Comparison of dexamethasone inhibitory effect in equine and human neutrophils. Neutrophils were isolated from the blood of 6 healthy horses and 4 healthy human volunteers and were incubated for 5 h with or without lipopolysaccharide (LPS; 100 ng/mL) alone or combined with dexamethasone (DEX) at 10^−6^ M. Following culture, mRNA expression of pro-inflammatory cytokines (IL-1β, TNF-α and IL-8; A, B and C) was quantified by qPCR. Absolute values were corrected using GAPDH as a reference gene. Dexamethasone percentage of inhibition on LPS-induced mRNA expression was compared between both species. A 100% inhibition means that mRNA expression returned to unstimulated cell basal mRNA level. Bars represent means + SEM. *Significant effect of treatment between cell populations (p = 0.013).

To evaluate possible interspecies difference, we compared the effects of dexamethasone between human and equine neutrophils. Dexamethasone had a similar inhibitory effect in both human and equine neutrophils for IL-1β and IL-8 (Figure 5.A and 5.C). However, a stronger transrepressing effect on TNF-α mRNA expression was observed in equine neutrophils when compared to human neutrophils (p = 0.013; Figure 5B).

**Figure 6:**
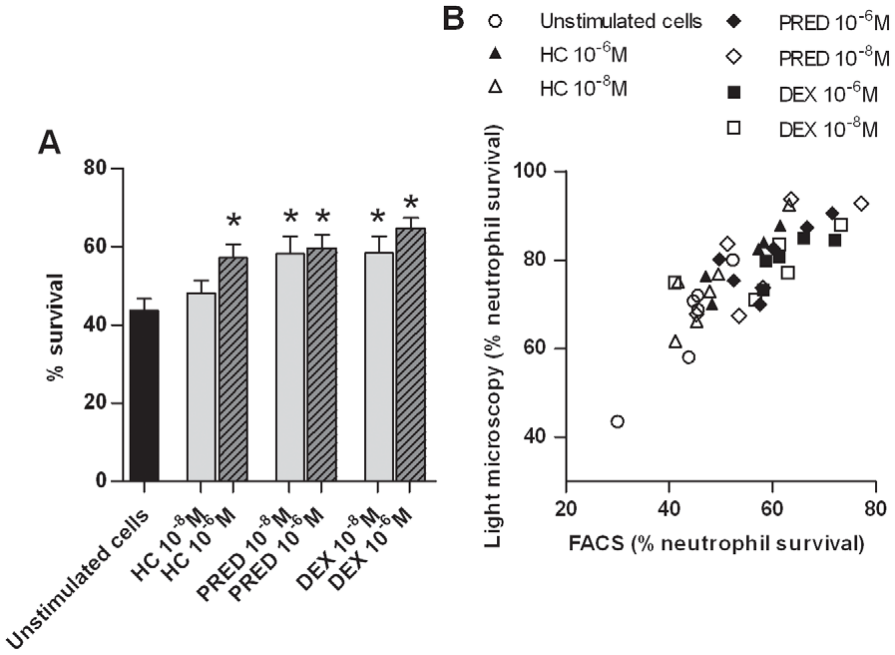
Glucocorticoids increase survival in equine peripheral blood neutrophils. A. Freshly isolated equine neutrophils (n = 6) were incubated for 20 h in the absence or presence of hydrocortisone (HC), prednisolone (PRED), dexamethasone (DEX) at 10^−6^ M and 10^−8^ M. The number of viable cells was assessed by flow cytometry using APC Annexin V/7-AAD staining. APC Annexin V and 7-AAD negative cells were considered as viable cells. Bars represent means + SEM. *Significant effect of treatment over unstimulated cells (p≤0.001). B. The percentage of viable cells was assessed by flow cytometry using APC Annexin V/7-AAD staining (x axis) and by light microscopy (y axis) and analyzed using a simple linear regression (linear regression slope R^2^ = 0.67, p<0.0001).

Finally, to ensure that GCs delayed equine neutrophil apoptosis as seen in humans [6], [7] neutrophil survival was assessed by flow cytometry and light microscopy. Indeed, hydrocortisone (10^−6^ M), prednisolone and dexamethasone (both at 10^−6^ M and 10^−8^ M) significantly increased the survival in equine neutrophils as assessed by flow cytometry (p≤0.001; Figure 6.A). A strong linear relationship was documented between values obtained by flow cytometry and by light microscopy (Figure 6.B; r^2^ = 0.67, p<0.0001, n = 6).

## Discussion

In the present study, we investigated the believed relative GC-insensitivity of neutrophils by comparing the response of LPS-stimulated peripheral blood neutrophils and neutrophil-depleted leukocytes from healthy horses and human volunteers to 3 commonly prescribed drugs. We found that GCs exert gene-specific, cortico-specific and concentration-dependent genomic effects within each cell population. Also neutrophils were found to be at least as responsive as other blood leukocytes to GCs in both species. Taken together, these results challenge the concept that the poor response to corticotherapy observed in some chronic neutrophilic diseases is due to an inherent attenuated response of neutrophils to GCs.

The effects of GCs occur through binding and activation of the cytoplasmic GR-α isoform. After translocation to the nucleus, GC-GR-α complex interferes with pro- and anti-inflammatory genes via transcription factors such as NF-κB [26], [27], [28]. GCs have also been shown to inhibit inflammation without requiring gene interaction, in a short-time, generally within minutes. These non-genomic effects of GCs on neutrophils were reported to be lesser than other inflammatory cells in an earlier study [5]. This led to the assertion that neutrophils are inherently less sensitive to GCs compared to other leukocytes. However, our group recently showed that dexamethasone potently reduced the oxidative respiratory burst in equine neutrophils at a physiologic dose as also used by Schleimer and colleagues when studying human neutrophils (*i.e.* 10^−6^ M) [5]. Furthermore, a marked reduction of reactive oxygen species generation was observed in both neutrophils and mononuclear cells following intravenous administration of dexamethasone in human healthy subjects [29]. Taken together, these findings question the hypothesis of an attenuated response to GCs through the non-genomic pathway in neutrophils.

We thus investigated herein the genomic effects of GCs in neutrophils and neutrophil-depleted leukocytes harvested from the same equine and human subjects. We observed a decreased expression of pro-inflammatory cytokines by GCs in neutrophils from both species. These findings are in agreement with, and extent the previous findings that GCs inhibits pro-inflammatory gene expression in neutrophils in different species [14], [15], [16], [17], [18], [19], [20]. This effect was also observed in neutrophil-depleted leukocytes however to a lesser extent for some genes (IL-1β and TNF-α). Moreover, dexamethasone down-regulated LPS-induced pro-inflammatory cytokines to their basal level of mRNA expression in human neutrophils while a residual effect of LPS still persisted in neutrophil-depleted leukocytes. These findings do not necessary imply stronger effects on neutrophils, as neutrophil-depleted leukocytes may have different kinetics for GC effects on pro-inflammatory gene expression. Nevertheless, the expression of glutamine synthetase, a glucocorticoid-inducible gene [30], [31], was increased by GCs to a greater extent in neutrophils compared to neutrophil-depleted leukocytes. In overall, GCs exerted effects of similar magnitude between cell populations in both human and equine subjects. We also found that equine and human neutrophils responded similarly to GCs. To our knowledge, this is the first report comparing the GC effects between equine and human neutrophils.

Dexamethasone is considered to be approximately 6–12 times and 25 times more potent than prednisolone and hydrocortisone, respectively. These conclusions are based on *in vivo* human and animal studies and *in vitro* experiments using a transactivation assay of cells transfected with human GR [32]. In the present study, we observed that dexamethasone was only twice as potent as hydrocortisone at inhibiting or transactivating mRNA expression. Dosages may have contributed to these apparent differences, as GC concentrations used to calculate GC potency in previous reports were far below those we studied. Also, the experimental settings we used differed from those of Grossmann and colleagues [32], possibly also contributing to these differences. Our group recently reported a significant improvement in pulmonary function mediated by prednisolone and dexamethasone in heaves-affected horses, a disease that share many features of human asthma [33]. To achieve equivalent potencies, doses for both drugs administrated were calculated based on their oral bioavaibility and reported ratios [32]. Yet, dexamethasone was found more effective than prednisolone in treatment of heaves-affected horses. Balance between genomic and non-genomic effects [34], influence of cell type, various affinities for the GR [32] may also explain differences observed in reported relative potencies of GCs.

The concentrations studied here were chosen because they are reached systemically at dosage commonly prescribed for dexamethasone in horses [35] and other GCs in humans [36], [37]. In addition, GR saturation threshold ranges from 10^−8^ M to 10^−6^ M, as reported in a study performed on purified rat peritoneal mast cells [38]. A maximal effect was not reached for all genes at 10^−8^ M in the present study however. For instance, hydrocortisone and prednisolone had a lesser transactivating effect on glutamine synthetase mRNA expression at 10^−8^ M, whereas a maximal potency at inhibiting IL-1β and IL-8 gene expression was already present at this concentration. This highlights the finely tuned regulation of the genomic pathway of GCs in neutrophils.

We chose LPS as the agonist to stimulate cells, as humans and horses are highly LPS-sensitive species [39], [40], [41]. Unexpectedly, the responsiveness to LPS we observed differed between cell populations of humans and horses. Equine neutrophils were shown to be more responsive to LPS than equine neutrophil-depleted leukocytes and, inversely for human cell populations. Human monocytes were shown to express LPS-signaling cell surface receptors Toll-like receptor 2 (TLR2), TLR4, and CD14 at higher levels than neutrophils [42]. In addition, human neutrophils were shown to express 10 to 20-fold less cytokines mRNA than monocytes [1]. In our study, the percentage of monocytes in horses and human volunteers were 15.0±6.7 and 27.5±8.7 (mean ± SD) respectively. We hypothesized that variations in the percentage of monocytes within neutrophil-depleted leukocytes contributed to the different response patterns towards LPS in both species. LPS had no effect on GR-α mRNA expression in equine neutrophils and neutrophil-depleted leukocytes, while LPS combined with dexamethasone down-regulated GR-α mRNA expression. Nevertheless, LPS was reported to reduce GR mRNA expression in lung tissue in a rat model of acute lung injury model [43]. Thus, a decrease in GR expression in neutrophils in LPS associated diseases may lead to loss of GC efficacy and corticoresistance.

Human and equine neutrophils participate to the immunomodulation of acute phase inflammation by the release of potent pro-inflammatory cytokines and chemokines, such as IL-1β, TNF-α, IL-6, IL-8 and macrophage inflammatory protein-2 [1], [21], [44]. A prolonged activation through a delayed apoptosis of neutrophils may contribute to sustained inflammation and result in massive damage in surrounding tissues. Several *in vitro* studies have shown that GCs increase human neutrophil survival by delaying apoptosis [6], [7]. Although molecular mechanisms implicated in these responses remain poorly understood, studies suggested that both genomic and non-genomic pathways may be involved. Various mechanisms for GC-mediated inhibition of apoptosis have been proposed including up-regulation of anti-apoptotic Bcl-2 and IAPs family members [45]. For instance, it has been demonstrated that dexamethasone induces survival and enhances Mcl-1, an inhibitor of Bax, a pro-apoptotic Bcl-2 family member, via PI3K and p38 MAPK kinases in human neutrophils [46]. Results of our present study indicate that, as in human species, GCs also enhances equine neutrophil survival. In agreement with our observations, Cox and colleagues also reported that GC-induced survival was not associated with cell activation as assessed by IL-8 release in culture supernatant over 24 h and superoxide production. These findings differ from data obtained using neutrophils stimulated with LPS, where decreased apoptosis was associated with sustained pro-inflammatory cytokine production [47]. Future studies assessing the genomic and non-genomic effects of a combination of both agonists on fresh and 24 h-cultured neutrophils are of interest. Indeed, GCs are used therapeutically at times when stimuli such as LPS might also be present.

Results of our study do not support an attenuated response of neutrophils to GCs through the genomic pathway. Although GC effects on anti-inflammatory gene expression in immune cells were not assessed, this may warrant further investigation as a recent *in vitro* study reported that dexamethasone down-regulated secreted IL-1receptor antagonist more efficiently than IL-β in human TNF-α-stimulated neutrophils, inducing a pro-inflammatory shift in the cytokine balance [48]. This finding may contribute to GC-resistance observed in severe asthma or COPD. However, there are some highly GC-responsive neutrophilic conditions. For instance, GC are very efficient drugs in human Sweet’s syndrome, an acute febrile dermatosis, characterized by a diffuse infiltration of mature neutrophils in the upper dermis [49]. In horses, GCs are commonly used for the treatment of heaves [50]. Despite a significant improvement in clinical signs, airway neutrophilia, a characteristic finding, persists after GC administration [50], [51], [52]. In light of our findings, an increase in neutrophil survival mediated by GCs is more likely contributing to persistence of these cells in tissues than an attenuated response of neutrophils to these drugs.

In conclusion, results from our study indicate that glucocorticoids exert genomic effects of similar magnitude in neutrophils and other blood leukocytes in both horses and humans. We therefore speculate that the poor response to corticotherapy observed in patients with severe asthma or COPD cannot be explained by a relative lack of inhibition of these drugs on pro-inflammatory cytokines expression in neutrophils. GC-resistant inflammatory diseases have been extensively studied during the last decade and several distinct molecular mechanisms contributing to decrease anti-inflammatory effects of GCs have now been identified. Among these mechanisms, an increase in the inactive isoform β expression of GR (GR-β) in GC-resistant inflammatory diseases has been proposed [8], [53]. Neutrophils highly express GR-β compared to other cell types [54]. Moreover, Hamid and colleagues recently suggested that an environment in which there is an increased level of interleukin-17 (IL-17) may influence GR-β signaling and steroid-responsiveness in asthmatic patients [55]. Future studies addressing potential direct or indirect effects of IL-17 on neutrophil GC-sensitivity would be of interest.

## Supporting Information

Table S1 **Sequences of primer pairs used for qPCR analysis.**
(DOC)

## References

[1] Cassatella MA (1999) Neutrophil-derived proteins: selling cytokines by the pound. Adv Immunol 73: 369–509. 10399011 10.1016/s0065-2776(08)60791-9

[2] Filep JG, El Kebir D (2009) Neutrophil apoptosis: a target for enhancing the resolution of inflammation. J Cell Biochem 108: 1039–1046. 19760640 10.1002/jcb.22351

[3] Schleimer RP (1990) Effects of glucocorticosteroids on inflammatory cells relevant to their therapeutic applications in asthma. Am Rev Respir Dis 141: S59–69. 2178515

[4] Schleimer RP (2004) Glucocorticoids suppress inflammation but spare innate immune responses in airway epithelium. Proc Am Thorac Soc 1: 222–230. 16113438 10.1513/pats.200402-018MS

[5] Schleimer RP, Freeland HS, Peters SP, Brown KE, Derse CP (1989) An assessment of the effects of glucocorticoids on degranulation, chemotaxis, binding to vascular endothelium and formation of leukotriene B4 by purified human neutrophils. J Pharmacol Exp Ther 250: 598–605. 2547940

[6] Cox G (1995) Glucocorticoid treatment inhibits apoptosis in human neutrophils. Separation of survival and activation outcomes. J Immunol 154: 4719–4725. 7722324

[7] Meagher LC, Cousin JM, Seckl JR, Haslett C (1996) Opposing effects of glucocorticoids on the rate of apoptosis in neutrophilic and eosinophilic granulocytes. J Immunol 156: 4422–4428. 8666816

[8] Barnes PJ, Adcock IM (2009) Glucocorticoid resistance in inflammatory diseases. Lancet 373: 1905–1917. 19482216 10.1016/S0140-6736(09)60326-3

[9] Ordonez CL, Shaughnessy TE, Matthay MA, Fahy JV (2000) Increased neutrophil numbers and IL-8 levels in airway secretions in acute severe asthma: Clinical and biologic significance. Am J Respir Crit Care Med 161: 1185–1190. 10764310 10.1164/ajrccm.161.4.9812061

[10] Wenzel SE, Schwartz LB, Langmack EL, Halliday JL, Trudeau JB, et al (1999) Evidence that severe asthma can be divided pathologically into two inflammatory subtypes with distinct physiologic and clinical characteristics. Am J Respir Crit Care Med 160: 1001–1008. 10471631 10.1164/ajrccm.160.3.9812110

[11] Wenzel SE, Szefler SJ, Leung DY, Sloan SI, Rex MD, et al (1997) Bronchoscopic evaluation of severe asthma. Persistent inflammation associated with high dose glucocorticoids. Am J Respir Crit Care Med 156: 737–743. 9309987 10.1164/ajrccm.156.3.9610046

[12] Yang IA, Fong KM, Sim EH, Black PN, Lasserson TJ (2007) Inhaled corticosteroids for stable chronic obstructive pulmonary disease. Cochrane Database Syst Rev: CD002991. 10.1002/14651858.CD002991.pub2 17443520

[13] Barnes PJ (2007) New molecular targets for the treatment of neutrophilic diseases. J Allergy Clin Immunol 119: 1055–1062; quiz 1063–1054. 10.1016/j.jaci.2007.01.015 17353033

[14] al-Mokdad M, Shibata F, Takano K, Nakagawa H (1998) Differential production of chemokines by phagocytosing rat neutrophils and macrophages. Inflammation 22: 145–159. 9561925 10.1023/a:1022383922039

[15] Strandberg K, Blidberg K, Sahlander K, Palmberg L, Larsson K (2010) Effect of formoterol and budesonide on chemokine release, chemokine receptor expression and chemotaxis in human neutrophils. Pulm Pharmacol Ther 23: 316–323. 20307681 10.1016/j.pupt.2010.03.004

[16] Nupponen I, Repo H, Kari A, Pohjavuori M, Andersson S (2002) Early dexamethasone decreases expression of activation markers on neutrophils and monocytes in preterm infants. Acta Paediatr 91: 1200–1207. 12463319 10.1080/080352502320777432

[17] Wertheim WA, Kunkel SL, Standiford TJ, Burdick MD, Becker FS, et al (1993) Regulation of neutrophil-derived IL-8: the role of prostaglandin E2, dexamethasone, and IL-4. J Immunol 151: 2166–2175. 8345201

[18] Burton JL, Kehrli ME Jr, Kapil S, Horst RL (1995) Regulation of L-selectin and CD18 on bovine neutrophils by glucocorticoids: effects of cortisol and dexamethasone. J Leukoc Biol 57: 317–325. 7531748 10.1002/jlb.57.2.317

[19] Weber PS, Toelboell T, Chang LC, Tirrell JD, Saama PM, et al (2004) Mechanisms of glucocorticoid-induced down-regulation of neutrophil L-selectin in cattle: evidence for effects at the gene-expression level and primarily on blood neutrophils. J Leukoc Biol 75: 815–827. 14761937 10.1189/jlb.1003505

[20] Lecoq L, Vincent P, Lavoie-Lamoureux A, Lavoie JP (2009) Genomic and non-genomic effects of dexamethasone on equine peripheral blood neutrophils. Vet Immunol Immunopathol 128: 126–131. 19036458 10.1016/j.vetimm.2008.10.303

[21] Joubert P, Silversides DW, Lavoie JP (2001) Equine neutrophils express mRNA for tumour necrosis factor-alpha, interleukin (IL)-1beta, IL-6, IL-8, macrophage-inflammatory-protein-2 but not for IL-4, IL-5 and interferon-gamma. Equine Vet J 33: 730–733. 11770998 10.2746/042516401776249246

[22] Lavoie-Lamoureux A, Beauchamp G, Quessy S, Martin JG, Lavoie JP (2012) Systemic inflammation and priming of peripheral blood leukocytes persist during clinical remission in horses with heaves. Vet Immunol Immunopathol 146: 35–45. 22342218 10.1016/j.vetimm.2012.01.020

[23] Haslett C, Guthrie LA, Kopaniak MM, Johnston RB Jr, Henson PM (1985) Modulation of multiple neutrophil functions by preparative methods or trace concentrations of bacterial lipopolysaccharide. Am J Pathol 119: 101–110. 2984939 PMC1888083

[24] Lavoie-Lamoureux A, Moran K, Beauchamp G, Mauel S, Steinbach F, et al (2010) IL-4 activates equine neutrophils and induces a mixed inflammatory cytokine expression profile with enhanced neutrophil chemotactic mediator release ex vivo. Am J Physiol Lung Cell Mol Physiol 299: L472–482. 20639353 10.1152/ajplung.00135.2009

[25] Fialkow L, Fochesatto Filho L, Bozzetti MC, Milani AR, Rodrigues Filho EM, et al (2006) Neutrophil apoptosis: a marker of disease severity in sepsis and sepsis-induced acute respiratory distress syndrome. Crit Care 10: R155. 17092345 10.1186/cc5090 PMC1794458

[26] Barnes PJ (2011) Glucocorticosteroids: current and future directions. Br J Pharmacol 163: 29–43. 21198556 10.1111/j.1476-5381.2010.01199.x PMC3085866

[27] Nicolaides NC, Galata Z, Kino T, Chrousos GP, Charmandari E (2010) The human glucocorticoid receptor: molecular basis of biologic function. Steroids 75: 1–12. 19818358 10.1016/j.steroids.2009.09.002 PMC2813911

[28] Pujols L, Mullol J, Picado C (2007) Alpha and beta glucocorticoid receptors: relevance in airway diseases. Curr Allergy Asthma Rep 7: 93–99. 17437678 10.1007/s11882-007-0005-3

[29] Dandona P, Mohanty P, Hamouda W, Aljada A, Kumbkarni Y, et al (1999) Effect of dexamethasone on reactive oxygen species generation by leukocytes and plasma interleukin-10 concentrations: a pharmacodynamic study. Clin Pharmacol Ther 66: 58–65. 10430110 10.1016/S0009-9236(99)70054-8

[30] Chandrasekhar S, Souba WW, Abcouwer SF (1999) Identification of glucocorticoid-responsive elements that control transcription of rat glutamine synthetase. Am J Physiol 276: L319–331. 9950895 10.1152/ajplung.1999.276.2.L319

[31] Gaunitz F, Heise K, Schumann R, Gebhardt R (2002) Glucocorticoid induced expression of glutamine synthetase in hepatoma cells. Biochem Biophys Res Commun 296: 1026–1032. 12200152 10.1016/s0006-291x(02)02044-2

[32] Grossmann C, Scholz T, Rochel M, Bumke-Vogt C, Oelkers W, et al (2004) Transactivation via the human glucocorticoid and mineralocorticoid receptor by therapeutically used steroids in CV-1 cells: a comparison of their glucocorticoid and mineralocorticoid properties. Eur J Endocrinol 151: 397–406. 15362971 10.1530/eje.0.1510397

[33] Leclere M, Lefebvre-Lavoie J, Beauchamp G, Lavoie JP (2010) Efficacy of oral prednisolone and dexamethasone in horses with recurrent airway obstruction in the presence of continuous antigen exposure. Equine Vet J 42: 316–321. 20525049 10.1111/j.2042-3306.2009.00022.x

[34] Buttgereit F, Brand MD, Burmester GR (1999) Equivalent doses and relative drug potencies for non-genomic glucocorticoid effects: a novel glucocorticoid hierarchy. Biochem Pharmacol 58: 363–368. 10423179 10.1016/s0006-2952(99)00090-8

[35] Grady JA, Davis EG, Kukanich B, Sherck AB (2010) Pharmacokinetics and pharmacodynamics of dexamethasone after oral administration in apparently healthy horses. Am J Vet Res 71: 831–839. 20594087 10.2460/ajvr.71.7.831

[36] Czock D, Keller F, Rasche FM, Haussler U (2005) Pharmacokinetics and pharmacodynamics of systemically administered glucocorticoids. Clin Pharmacokinet 44: 61–98. 15634032 10.2165/00003088-200544010-00003

[37] Xu J, Winkler J, Derendorf H (2007) A pharmacokinetic/pharmacodynamic approach to predict total prednisolone concentrations in human plasma. J Pharmacokinet Pharmacodyn 34: 355–372. 17318442 10.1007/s10928-007-9050-8

[38] Walajtys-Rode E, Dabrowski A, Grubek-Jaworska H, Machnicka B, Droszcz W (1988) Binding of dexamethasone and its effect on histamine release from rat mast cells. Int J Immunopharmacol 10: 925–930. 2463972 10.1016/0192-0561(88)90038-0

[39] Burrows GE (1981) Dose-response of ponies to parenteral Escherichia coli endotoxin. Can J Comp Med 45: 207–210. 7020894 PMC1320155

[40] Berczi I, Bertok L, Bereznai T (1966) Comparative studies on the toxicity of Escherichia coli lipopolysaccharide endotoxin in various animal species. Can J Microbiol 12: 1070–1071. 5339644 10.1139/m66-143

[41] Palsson-McDermott EM, O’Neill LA (2004) Signal transduction by the lipopolysaccharide receptor, Toll-like receptor-4. Immunology 113: 153–162. 15379975 10.1111/j.1365-2567.2004.01976.x PMC1782563

[42] Sabroe I, Jones EC, Usher LR, Whyte MK, Dower SK (2002) Toll-like receptor (TLR)2 and TLR4 in human peripheral blood granulocytes: a critical role for monocytes in leukocyte lipopolysaccharide responses. J Immunol 168: 4701–4710. 11971020 10.4049/jimmunol.168.9.4701

[43] Wang XQ, Zhou X, Zhou Y, Rong L, Gao L, et al (2008) Low-dose dexamethasone alleviates lipopolysaccharide-induced acute lung injury in rats and upregulates pulmonary glucocorticoid receptors. Respirology 13: 772–780. 18657064 10.1111/j.1440-1843.2008.01344.x

[44] Cassatella MA (1995) The production of cytokines by polymorphonuclear neutrophils. Immunol Today 16: 21–26. 7880385 10.1016/0167-5699(95)80066-2

[45] Saffar AS, Ashdown H, Gounni AS (2011) The molecular mechanisms of glucocorticoids-mediated neutrophil survival. Curr Drug Targets 12: 556–562. 21504070 10.2174/138945011794751555 PMC3267167

[46] Saffar AS, Dragon S, Ezzati P, Shan L, Gounni AS (2008) Phosphatidylinositol 3-kinase and p38 mitogen-activated protein kinase regulate induction of Mcl-1 and survival in glucocorticoid-treated human neutrophils. J Allergy Clin Immunol 121: 492–498 e410. 10.1016/j.jaci.2007.10.003 18036649

[47] Colotta F, Re F, Polentarutti N, Sozzani S, Mantovani A (1992) Modulation of granulocyte survival and programmed cell death by cytokines and bacterial products. Blood 80: 2012–2020. 1382715

[48] Langereis JD, Oudijk EJ, Schweizer RC, Lammers JW, Koenderman L, et al (2011) Steroids induce a disequilibrium of secreted interleukin-1 receptor antagonist and interleukin-1beta synthesis by human neutrophils. Eur Respir J 37: 406–415. 20650986 10.1183/09031936.00170409

[49] Cohen (2007) Sweet’s syndrome – a comprehensive review of an acute febrile neutrophilic dermatosis. Orphanet Journal of Rare Diseases. 10.1186/1750-1172-2-34 PMC1963326 17655751

[50] Leclere M, Lavoie-Lamoureux A, Lavoie JP (2011) Heaves, an asthma-like disease of horses. Respirology 16: 1027–1046. 21824219 10.1111/j.1440-1843.2011.02033.x

[51] Lavoie JP, Pasloske K, Joubert P, Cordeau ME, Mancini J, et al (2006) Lack of clinical efficacy of a phosphodiesterase-4 inhibitor for treatment of heaves in horses. J Vet Intern Med 20: 175–181. 16496938 10.1892/0891-6640(2006)20[175:loceoa]2.0.co;2

[52] Lavoie JP, Leguillette R, Pasloske K, Charette L, Sawyer N, et al (2002) Comparison of effects of dexamethasone and the leukotriene D4 receptor antagonist L-708,738 on lung function and airway cytologic findings in horses with recurrent airway obstruction. Am J Vet Res 63: 579–585. 11939323 10.2460/ajvr.2002.63.579

[53] Barnes PJ (2010) Mechanisms and resistance in glucocorticoid control of inflammation. J Steroid Biochem Mol Biol 120: 76–85. 20188830 10.1016/j.jsbmb.2010.02.018

[54] Strickland I, Kisich K, Hauk PJ, Vottero A, Chrousos GP, et al (2001) High constitutive glucocorticoid receptor beta in human neutrophils enables them to reduce their spontaneous rate of cell death in response to corticosteroids. J Exp Med 193: 585–593. 11238589 10.1084/jem.193.5.585 PMC2193396

[55] Vazquez-Tello A, Semlali A, Chakir J, Martin JG, Leung DY, et al (2010) Induction of glucocorticoid receptor-beta expression in epithelial cells of asthmatic airways by T-helper type 17 cytokines. Clin Exp Allergy 40: 1312–1322. 20545708 10.1111/j.1365-2222.2010.03544.x

