# Supplementary material for: Neutrophils Are Not Less Sensitive Than Other Blood Leukocytes to the Genomic Effects of Glucocorticoids
Source: PLoS One. 2012 Sep 12;7(9):e44606. doi: 10.1371/journal.pone.0044606 (PMC3440353; doi:10.1371/journal.pone.0044606)
Supplement: Table S1 — Sequences of primer pairs used for qPCR analysis. (DOC) [file pone.0044606.s001.doc]

**Table S1. Sequences of primer pairs used for qPCR analysis.**

| **Species** | **Gene Id** | **Forward sequence (5’–3’)** | **Reverse sequence (5’–3’)** |
| --- | --- | --- | --- |
| *Equus caballus* | *GAPDH* | AAGTGGATATTGTCGCCATCAAT | AACTTGCCATGGGTGGAATC |
|  | *IL-1β* | GACTGACAAGATACCTGTGGCCT | AGACAACAGTGAAGTGCAGCCT |
|  | *IL-8* | GCAGACCTCAGCTCCGTTGAC | CTTTCTGCAGCTCTGTGTGAAG |
|  | *TNF-α* | CTTGTGCCTCAGCCTCTTCTCCTTC | TGATGGCAGAGAGGAGGTTGA |
|  | *Glutamine synthetase* | ACTGGATTCCACGAAACCTCCAAC | GCTGCAAGTCTAGTCCGCTTAGTT |
|  | *GR-α* | GAAGGAAACTCCAGCCAGAAC | CTGATTGGTGATGATTTCAGCTA |
| *Homo sapiens* | *GAPDH* | GTGAAGGTCGGAGTCAACGGATTT | TTGACGGTGCCATGGAATTTGC |
|  | *IL-1β* | ACCTGAGCTCGCCAGTGAAATGAT | TGGTCGGAGATTCGTAGCTGGAT |
|  | *IL-8* | CCTTCCTGATTTCTGCAGCTCTGT | TTGGGGTGGAAAGGTTTGGAGT |
|  | *TNF-α* | TGACAAGCCTGTAGCCCATGTT | GTTATCTCTCAGCTCCACGCCATT |
